# Alterations in the Cell Wall of *Rhodococcus biphenylivorans* Under Norfloxacin Stress

**DOI:** 10.3389/fmicb.2020.554957

**Published:** 2020-10-06

**Authors:** Yangyang Jia, Chungui Yu, Jiahui Fan, Yulong Fu, Zhe Ye, Xiaoguang Guo, Ying Xu, Chaofeng Shen

**Affiliations:** ^1^Department of Environmental Engineering, College of Environmental and Resource Sciences, Zhejiang University, Hangzhou, China; ^2^State Key Laboratory of Microbial Metabolism, School of Life Sciences and Biotechnology, Shanghai Jiao Tong University, Shanghai, China; ^3^Zhejiang Provincial Key Laboratory for Water Pollution Control and Environmental Safety, Hangzhou, China

**Keywords:** *Rhodococcus biphenylivorans*, cell wall, viable but non-culturable state, resuscitation, environmental stresses, peptidoglycan

## Abstract

Many microorganisms can enter a viable but non-culturable (VBNC) state under various environmental stresses, while they can also resuscitate when the surroundings turn to suitable conditions. Cell walls play a vital role in maintaining cellular integrity and protecting cells from ambient threats. Here, we investigated the alterations in the cell wall of *Rhodococcus biphenylivorans* TG9 at VBNC state under norfloxacin stress and then at resuscitated state in fresh lysogeny broth medium. Electron microscopy analyses presented that TG9 in the VBNC state had a thicker and rougher cell wall than that in exponential phase or resuscitated state. Meanwhile, the results from infrared spectroscopy also showed that its VBNC state has different peptidoglycan structures in the cell wall. Moreover, in the VBNC cells the gene expressions related to cell wall synthesis and remodeling maintain a relatively high level. It indicates that the morphological variations of TG9 at the VBNC state might result from kinetic changes in the cell wall synthesis and remodeling. As a consequence, the alterations in the cell wall of VBNC TG9 may somewhat account for its tolerance mechanisms to antibiotic treatment.

## Introduction

Microorganisms are constantly exposed to a variety of environmental stresses, including unsuitable temperature, nutrient deficiency, hypoxia, or exposure to toxic compounds, such as heavy metals and antibiotics (Matilla, 2018). Bacteria have developed several mechanism to survive during stress (Kim et al., 2018). One of these strategies involves entering a viable but non-culturable (VBNC) state (Colwell, 1996). Bacteria in the VBNC state cannot be cultivated, but maintain their viability, have a lowered metabolism, and their cultivability can be restored by changing the conditions (Boaretti et al., 2003; Lleò et al., 2007). Currently, over 100 bacterial species have been confirmed to be able to enter the VBNC state (Pinto et al., 2013; Ayrapetyan et al., 2015; Fida et al., 2017), including *Vibrio cholerae*, *Escherichia coli*, *Micrococcus luteus*, and *Mycobacterium tuberculosis*, among others (Kamruzzaman et al., 2010; Oliver, 2010; Ayrapetyan et al., 2018). Since the majority of these bacterial species are pathogens, several studies have been carried out focusing on the infectious and pathogenic potential of bacterial species in the VBNC state (Cervero-Arago et al., 2019; Zhong et al., 2019).

Pollutant-degrading bacteria can also enter the VBNC state. Fida et al. (2017) reported that the number of colony forming units (CFU) of phenanthrene-degrading *Novosphingobium* sp. LH128 declined rapidly when LH128 was introduced into phenanthrene-containing soil due to entering a VBNC-like state. In our previous studies, a polychlorinated biphenyls (PCBs)-degrading strain of *Rhodococcus biphenylivorans* TG9 was isolated from PCB-contaminated sediment and could enter a VBNC state for 145 days when maintained in low nutrient and low temperature conditions (Su et al., 2015b). Entering the VBNC state can have a large impact on the pollutant-degrading ability of this bacterial species and may be the reason why efficient pollutant-degrading bacteria usually show low activity in environmental bioremediation (Su et al., 2015b; Ye et al., 2020). For example, we found that TG9 cells in the VBNC state had a low degradation efficiency of PCBs, and the degradation efficiency could be greatly improved only after the cells were resuscitated (Ye et al., 2020). Therefore, understanding the mechanism of the VBNC state is key for improving the degradation ability and practical application of this bacterial species.

In most bacterial cells, the cell wall acts as a stress-bearing structure that maintains cellular integrity, dictates cell shape, and provides mechanical strength for resistance against osmotic challenges (Huang et al., 2008; Turner et al., 2013). Alterations to the cell walls are a common feature of dormant bacteria (Rittershaus et al., 2013). For example, compared to vegetative cells, *Bacillus* spores have a thicker cell wall with changed cross-linking (Meador-parton and Popham, 2000). Persistent *Mycobacterium tuberculosis* possesses a thick outer layer to restrict the entry of rifampicin under hypoxic conditions (Jakkala and Ajitkumar, 2019). However, as a type of dormant cell, very limited information is available on changes of the cell wall in VBNC cells. When enter into the VBNC state, the rod-shaped cells usually turn into coccoid (Dong et al., 2019). Given that the cell wall is responsible for determining cell shape, these morphological changes may also result from changes in the cell wall. Furthermore, microbial populations exit dormancy in response to muropeptides of the cell wall (Dworkin and Shah, 2010). For example, VBNC cells can be reactivated by a type of lysozyme-like protein, namely resuscitation-promoting factors (Rpfs), which are able to lyse the cell walls peptidoglycan (Cohen-Gonsaud et al., 2005). The alteration of the cell wall appears to represent a bridge between entering the VBNC state and resuscitation, and may thus play a vital role in the maintenance and exit of the VBNC state.

Peptidoglycan is the main component of the cell wall of gram-positive bacteria, and the synthesis and remodeling of peptidoglycan are similar among different bacteria (Monteiro et al., 2018). Firstly, the monomer of peptidoglycan, glycan tetrapeptide, is synthesized in the cytoplasm, under the catalysis of the Mur ligase family (Madigan et al., 2011; Goncalves et al., 2019). Then, these monomers are transported outside the cell membrane and are linked by covalent bonds and cross-linked by amino acids to form long-chain peptidoglycan, catalyzed by GTases and dd-TPases (MraY, MurG, PBPs) and lipid II flippase (FtsW) (Typas et al., 2011). The cleavage of covalent bonds is required for attaching new monomers to the existing peptidoglycan sacculus. This process requires the catalysis of many peptidoglycan hydrolases (autolysins). Besides, the fragments removed from the sacculus can be reused for the peptidoglycan remodeling (Typas et al., 2011).

Here, we investigated the changes in the cell wall of *Rhodococcus biphenylivorans* TG9 cells entering the VBNC state and during resuscitation. We chose the antibiotic, norfloxacin, as a inducer, since the organic pollutant degrading bacterium might enter into dormancy state such as VBNC state under the stress of antibiotics in the organic polluted environment. Besides, the norfloxacin works by inhibiting DNA gyrase and topoisomerase IV, without damaging the cell walls. Changes in the physical properties, chemical structure, and expression levels of related genes in the cell wall of TG9 cells during this process were investigated to gain a detailed insight into the relationship between changes in the cell walls, VBNC cell formation, and resuscitation.

## Materials and Methods

### Bacterial Strain and Growth Conditions

The strain *Rhodococcus biphenylivorans* TG9^T^ (CGMCC 1.12975^T^) was used in this study (Su et al., 2015a). The strain was preserved in lysogeny broth (LB) with 15% glycerol (v/v) at −80°C. Prior to use, bacterial cells were grown in sterile LB medium on a shaker incubator (180 rpm) at 30°C for 24 h. The cells were then inoculated (1%, v/v) in LB medium under the same conditions until grown to the exponential phase (OD_600_ = 1).

### Induction of VBNC Cells

Norfloxacin (purity 98%, Bailingwei Technology Co., Ltd., China) was added to the exponential phase cells (OD_600_ = 1) at a final concentration of 64 mg/L. The cells were cultured in the dark on a shaker incubator (180 rpm) at 30°C for several days to induce the VBNC state. Triplicate experiments were performed. The flow cytometer (BD FACSMelody, United States) and LB agar plates were used to analyze each sample obtained at different times.

### Analysis of Viability and Culturability

The counts of culturable cell were determined using the standard plate count method according to Su et al. (2015b). Each sample was serially diluted 10-fold with 0.9% (w/v) physiological saline (sodium chloride solution) and incubated on LB agar at 30°C for 48 h. The culturable cell counts of *R. biphenylivorans* TG9 were calculated as counts per milliliter (CFU/mL). The viable cells number was measured using a flow cytometer. The 5-cyano-2, 3-ditolyl tetrazolium chloride (CTC) (Dojindo, Japan) staining was used to determine the viable cells. Bacteria in the VBNC state still have active respiration. CTC can be reduced by electron transport chains to give off red fluorescence whose intensity reflects the number of viable cells (Zhang et al., 2018). CTC solution was added to 500 μL of *R. biphenylivorans* TG9 at a final CTC concentration of 1 mM. Then the suspensions were mixed with 50 μL absolute counting beads (Thermo Scientific, United States). Before analysis, the cell suspensions were incubated at 37°C for 1 h in the dark. When the number of cultured cells had decreased to an undetectable level, the cells still viable were denoted as entering the VBNC state. The VBNC cells number was calculated as the viable cells number minus the culturable cells number (Chen et al., 2019).

### Resuscitation From the VBNC State of *R. biphenylivorans*

After centrifugation at 8000 *g* for 15 min, the VBNC cells were collected and washed twice with 0.9% (w/v) physiological saline. The cells were then resuspended in the same volume of LB medium on a shaker incubator (180 rpm) at 30°C for 72 h. All experiments were performed at least three times. A flow cytometer and LB agar plates were used to analyze each sample obtained at different times.

### Transmission Electron Microscopy (TEM)

Samples preparation for TEM was described previously (Chuang et al., 2015). The cells in the exponential phase, VBNC state, and after resuscitation were initially fixed with 2.5% glutaraldehyde in 0.1M phosphate buffer (PBS) overnight. The samples were then washed three times in PBS (15 min each time). Subsequently, the samples were fixed for 1 h with 1% OsO_4_ in PBS. After washing three times with PBS for 15 min each time, the samples were dehydrated using a graded ethanol series: 30, 50, 70, 80, 90, 95, and 100%, before transferring to absolute acetone and embedding in epoxy resin. The samples were sectioned and stained with 2% uranyl acetate in 50% ethanol and 1% alkaline lead citrate, each for 15 min. The stained sections were then viewed by TEM (Hitachi Model H-7650). The thickness of the cell walls was measured using ImageJ. A total of 50 bacterial cells were measured in each experiment. Each cell was measured three times, and we took the average.

### Scanning Electron Microscopy (SEM)

The preparation of samples for SEM was similar to that for TEM. The only difference was that the samples for SEM were dried with a critical point dryer (Hitachi Model HCP-2) after treatment with ethanol. The samples were then coated with gold-palladium and viewed by SEM (Hitachi Model SU-8010).

### Atomic Force Microscopy (AFM)

Intact bacteria were prepared according to Dover et al. (2015), with some modifications. The bacterial culture of exponential phase cells and VBNC cells were washed twice, and resuspended in PBS. The cells were immobilized on a poly-L-lysine-coated MICA for 20 min before washing to remove any unattached bacteria. The cells were then gently fixed using 1.5% glutaraldehyde for at least 10 min and washed with double distilled water (ddH_2_O) to remove glutaraldehyde. The samples were left to dry overnight at 25°C.

The images were observed by MultiMode AFM (Bruker icon, Santa Barbara, CA, United States), which equipped with a scan-asyst-air probe in scan-asyst-air mode. The engage setpoint was set to 0.05 V. Image and roughness analysis were carried out using NanoScope Analysis software (Bruker). A total of 10 bacterial cells were measured in each experiment.

### Fourier Transform Infrared Spectroscopy (FT-IR) Analysis

The cells in the exponential phase, VBNC state, and after resuscitation were harvested by centrifugation at 8000 *g* for 15 min and washed with PBS. The resulting pellets were washed with ddH_2_O and freeze-dried for infrared spectroscopy analysis. The peptidoglycan isolation from TG9 cells in the three different states was performed as described previously (Kuhner et al., 2014; Van der Aart et al., 2018), with some modifications. A total of 100 mL of the exponentially growing, VBNC, and resuscitation cultures were collected by centrifugation at 10,000 *g* for 20 min. The resulting pellets were boiled in 20 mL 0.25% (w/v) sodium dodecyl sulfate (SDS) in 0.1 M Tris-HCl (pH 6.8) for 30 min, then washed with distilled water thoroughly. The pellets were resuspended in 10 mL distilled water and sonicated for 30min. Then the samples were treated with 5 mL 0.1 M Tris-HCl (pH 6.8) containing 300 μg RNase (Diamond, Shanghai, China) and 75 μg DNase (BBI, Shanghai, China) for 60 min. Added 5 mL 50 μg/mL trypsin (Diamond, Shanghai, China) solution and incubated for 60 min. The enzymes were inactivated through boiling for 30 min and then washed twice with ddH_2_O. The teichoic acids were removed from the cell walls using 5 mL 1 M HCl and washed with ddH_2_O thoroughly. Peptidoglycan (PG) was resuspended in 2.5 mL 12.5 mM sodium dihydrogen-phosphate solution and digested by adding 250 μL 5 U/mL mutanolysin solution (Sigma-Aldrich, Germany) and 1 mL 10 mg/mL lysozyme (Diamond, Shanghai, China) for 16 h at 37°C. The enzymes were inactivated through boiling for 30 min and then the supernatant was collected by centrifugation at 10,000 *g* for 20 min. The samples were freeze-dried for infrared spectroscopy analysis.

Infrared spectroscopic was used to analyze the molecular structure of the whole cell and peptidoglycan. Briefly, 1 mg of samples were added to 100 mg of potassium bromide power and pressed into pellets for spectrometry analysis (Nicolet 6700 FT-IR; Thermo Fisher Scientific Inc.) (He et al., 2017).

### Real-Time Quantitative Reverse-Transcription PCR (RT-qPCR)

The expression of the four genes associated with cell wall was investigated by RT-qPCR according to Hung et al. (2013), with some modifications. The RNA samples were extracted by RNeasy Plus Mini Kit (Qiagen, Dusseldorf, Germany) according to the instructions provided by manufacturer. The cDNA was synthesized from 0.2 μg of total RNA by the PrimeScript^TM^ RT reagent Kit with gDNA Eraser (Takara, Japan). The primers were designed by Primer Blast and shown in Supplementary Table S1. The 16S rRNA gene was selected as the reference gene. The transcription levels of the 16S rRNA gene, *murA*, *ftsW*, *rpfE*, and *pknB* from *R. biphenylivorans* TG9 were examined. The PCR was performed in a total volume of 20 μL containing 0.8 μL of each primer (10 μM), 2 μL of cDNA, 10 μL of 2 × TB Green^®^ Premix Ex Taq^TM^ II (Takara, Japan), ROX Reference Dye II (Takara, Japan) and 6 μL of ddH_2_O. Amplification was performed using Applied Biosystems QuantStudio 3 and 5 Real-Time PCR Systems following the program: 95°C for 3 min; 40 cycles of 95°C (15 s), 52°C for 30 s, and 72°C for 30 s. The data was normalized to the expression of the 16S rRNA gene, and the transcript level of each gene was compared to its transcript level in the normal TG9 cells.

### Statistical Analysis

The data were analyzed by Student’s *t*-test and analysis of variance (ANOVA) in SPSS 20. And the significance level was set at *P* < 0.05.

## Results

### Induction and Resuscitation of VBNC State

**FIGURE 1:**
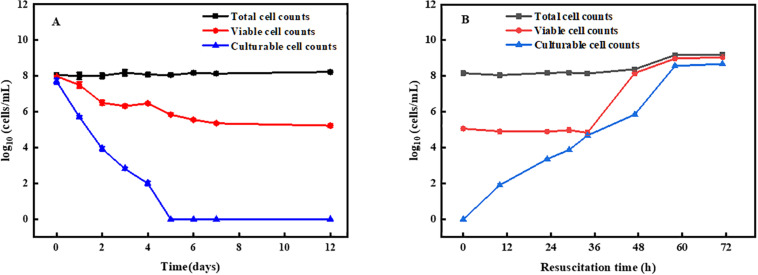
Induction and resuscitation of *R. biphenylivorans* TG9 cells in the VBNC state. **(A)** Entry into the VBNC state induced by treatment with norfloxacin, according to flow cytometry (■) (*O*) and the plate counting method (▲). **(B)** Resuscitation of VBNC cells.

After norfloxacin treatment, the number of VBNC bacterial cells was determined as the difference between the viable cell counts and the culturable cells counts using CTC and flow cytometry and the standard plate count methods (Guo et al., 2019). As shown in Figure 1A, the total cells counts remained 10^8^ cells/mL, while the number of living cells and cultured cells decreased gradually. After 5 days of treatment, the number of cultured cells was found to decrease to an undetectable level, and living cells counts remained 10^6^ cells/mL. At 7 days, the number of living cells decreased to 10^5^ cells/mL. After extending the treatment time, the number of living cells almost no longer decreased. These results suggested that approximately 10^5^ cells/mL could enter the VBNC state under the current condition and were norfloxacin-tolerant.

Moreover, VBNC cells were confirmed by resuscitation. The cells in the VBNC state were resuscitated in liquid medium. After culturing for 36 h, the number of resuscitated cells reached 10^5^ cells/mL, followed by a rapid increase (Figure 1B). The number of cells peaked at 60 h and remained stable. These results suggest that the increase in the number of cells after 36 h resulted from the proliferation of resuscitation cells, and that after 60 h, the cells entered the stationary phase (Su et al., 2015b).

### Change in Cell Wall Thickness

**FIGURE 2:**
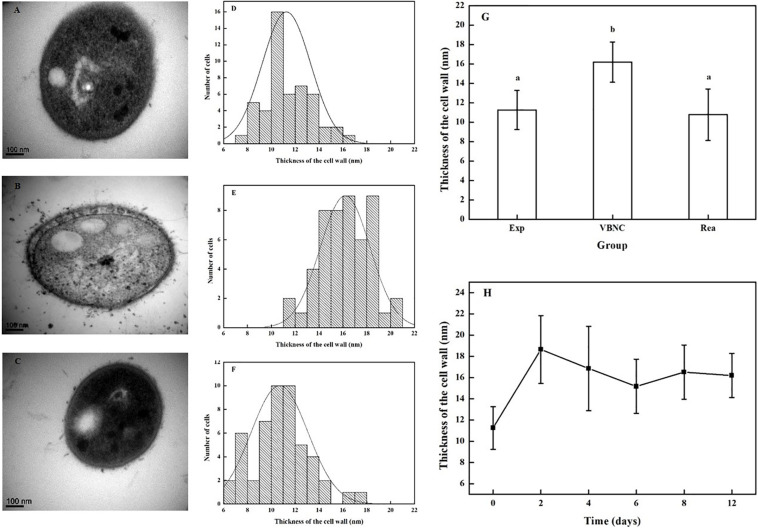
Change in the thickness of cells walls in *R. biphenylivorans* TG9 cells in the exponential phase, the VBNC state, and after resuscitation. TEM image of **(A)** exponential cells, **(B)** VBNC cells, and **(C)** resuscitated cells. The cell wall thickness distribution of **(D)** exponential cells (*n* = 50), **(E)** VBNC cells (*n* = 50), and **(F)** resuscitated cells (*n* = 50). **(G)** The thickness of the cell walls of *R. biphenylivorans* TG9 cells in the exponential phase, the VBNC state, and after resuscitation; Significant differences between means are indicated by different letters, *p* < 0.05. **(H)** Change in the thickness of cell walls during the induction of *R. biphenylivorans* into the VBNC state by treatment with norfloxacin.

Transmission electron microscopy of the VBNC cells (*n* = 50) in the transverse and longitudinal sections shown thick, rough, and strikingly uneven cell walls, unlike those of cells in the exponential phase (*n* = 50) and resuscitated cells (*n* = 50) (Figures 2A–C).

The thickness of the cell walls of the exponential phase cells, VBNC-state cells, and resuscitated cells were measured. As shown in Figures 2D–G, the mean cell wall thickness of the VBNC-state cells (16.20 ± 2.08 nm) was greater than that of the exponential phase cells (11.26 nm ± 2.01 nm). When the cells in the VBNC state were resuscitated, their cell wall became thinner (10.70 ± 2.36 nm). In addition, as shown in Figure 2H, the cell wall of *R. biphenylivorans* TG9 cells became thicker after 2 days of norfloxacin treatment, but remained almost constant during incubation.

### Change in Bacterial Surface Roughness

**FIGURE 3:**
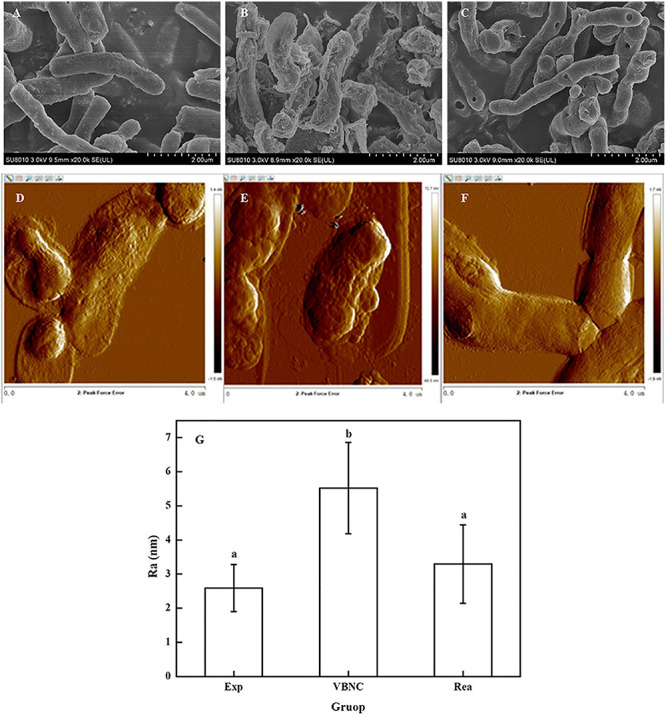
Change in the surface roughness of the cells walls of *R. biphenylivorans* TG9 cells in the exponential phase, the VBNC state, and after resuscitation. SEM image of **(A)** exponential cells, **(B)** VBNC cells, and **(C)** resuscitated cells. AFM image of **(D)** exponential cells, **(E)** VBNC cells, and **(F)** resuscitated cells. **(G)** The roughness of the cell walls of *R. biphenylivorans* TG9 cells in the exponential phase, the VBNC state, and after resuscitation; Significant differences between means are indicated by different letters, *p* < 0.05.

Scanning electron microscopy showed that the surface of the VBNC cells was rough and unevenly wrinkled, while that of the exponential phase cells and resuscitated cells was clear and smooth (Figures 3A–C). In addition, the surface of the cells in the exponential phase, VBNC state, and the resuscitated cells was examined using AFM. The AFM images revealed that the surface roughness values of the VBNC cells increased, while there was no indication that the bacterial cell envelope was damaged. Height images and roughness analysis of the cells’ surface revealed that the exponential phase cells were homogeneous and smooth, while the VBNC cells were rougher (Figures 3D–F). The average surface roughness of exponential phase cells was 2.59 ± 0.69 nm, while mean the surface roughness values for the VBNC cells were 5.52 ± 1.34 nm and 2.1-fold greater than the control. After VBNC cells were resuscitated, the average surface roughness value was 3.30 ± 1.15 nm (Figure 3G).

The results obtained from SEM and AFM confirmed that the morphology and surface roughness of the VBNC cells changed in response to antibiotic stress, unlike that of the exponential phase and resuscitated cells.

### FT-IR Analysis of Peptidoglycan

**FIGURE 4:**
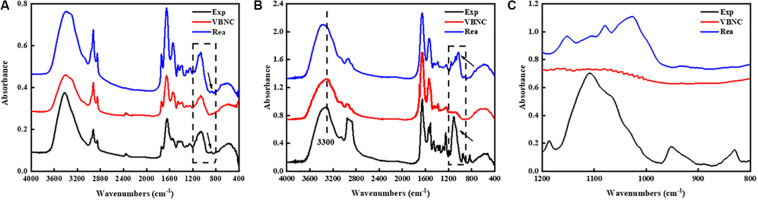
FT-IR spectrum of **(A)** *R. biphenylivorans* TG9 cells in the exponential phase, the VBNC state, and after resuscitation, the differences in the spectra of bacteria in different states in the 1200–800 cm^–1^ region which as shown by the arrow and vertical rectangle. **(B)** peptidoglycan and Characteristic peak of peptidoglycan were shown by the vertical rectangle and vertical dashed line, **(C)** peptidoglycan in 1200–800 cm^–1^ region.

As shown in Figure 4A, the FT-IR spectra of TG9 cells in different states showed small but important differences in the 1200–800 cm^–1^ region as shown by the arrow. The results suggested that the FT-IR spectral alterations might result from the compositional changes in the cell wall components.

To confirm whether the cell wall of VBNC-state cells changed, we isolated the main component of the cell wall, peptidoglycan (PG), from cells in the three states. FT-IR spectroscopy was then used to evaluate the compositional changes of the peptidoglycan. It has been reported that the window between 1200 and 900 cm^–1^ represented the C–O–C, C–O of ring vibrations of carbohydrates, which reveals the occurrence of carbohydrates and polysaccharides in the cell wall (Alvarez-Ordóñez and Prieto, 2012). Therefore, in the spectra of PG (Figure 4B), the bands in the region 1200–900 cm^–1^ were characteristic polysaccharide peaks. We have zoomed in on this region (Figure 4C).

The spectra of the VBNC state peptidoglycan was found to have important variations in the intensity and shape of several spectral bands compared to the spectra of peptidoglycan in exponential phase cells and resuscitated cells. Visible differences were also observed in the spectra near 1109 cm^–1^, which indicates that the peptidoglycan of VBNC cells and resuscitated cells was modified.

Besides, we have further analyzed the composition of the cell wall using LC-MS/MS according to Kuhner et al. (2014). The results were shown in the Supporting Information. Our results showed that the cell wall composition of VBNC-state cells did differ from that of exponential phase cells. However, due to the lack of the standard reference materials, we have not been able to analyze the results further.

### Transcription of Genes Related to the Synthesis and Remodeling of Cell Wall

**FIGURE 5:**
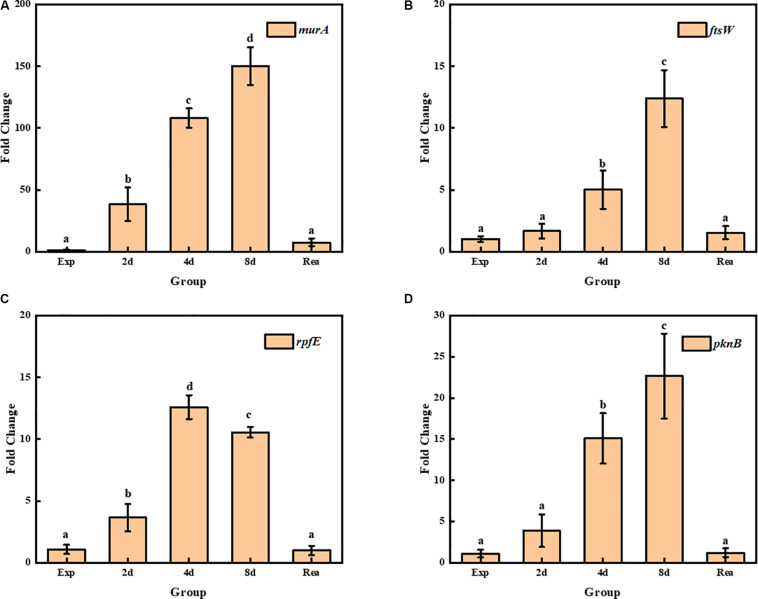
Transcript levels of **(A)** *murA*, **(B)** *ftsW*, **(C)** *rpfE*, and **(D)** *pknB* in exponential phase cells, VBNC cells and resuscitated cells; Significant differences between means are indicated by different letters, *p* < 0.05. The data was normalized to the expression of the 16S rRNA gene, and the transcript level of each gene was compared to its transcript level in the normal TG9 cells.

Four genes related to the synthesis and remodeling of cell wall were identified during TG9 entry into the VBNC state and after resuscitation. Several of the genes were found to be related to the synthesis and remodeling of cell wall, including *murA*, a UDP-*N*-acetylglucosamine 1-carboxyvinyl transferase gene, which catalyzes the first step of peptidoglycan synthesis (Boutte et al., 2016). The transcript level of *murA* in *R. biphenylivorans* was found to still maintain very high during the transition stage from normal cells to VBNC-state cells (Figure 5A). The *ftsW* gene encodes a peptidoglycan polymerase, which produces septal peptidoglycan during cell division. The transcript level of the *ftsW* gene was relatively increased 4 d after norfloxacin treatment (Figure 5B). Moreover, the expression of *rpfE*, encoding a muralytic enzyme for the lysis and remodeling of peptidoglycan (Sexton et al., 2015), was found to be relatively enhanced in VBNC cells comparing to the 16S gene (Figure 5C). While *pknB*, a protein kinase B gene, controlling the biosynthesis of peptidoglycan during cell growth (Alqaseer et al., 2019), was found to be relatively high expression during the TG9 entry into VBNC (Figure 5D).

## Discussion

The VBNC state formed by bacteria in the face of environmental stress has attracted more and more attention, especially in the field of pathogens. However, there are few reports about the VBNC state of degrading bacteria. In fact, entering VBNC state is one of the main problems of low degradation efficiency in practical application of degrading bacteria. In our previous study, we resuscitated a strain of *Rhodococcus biphenylivorans* TG9, which has a strong BP/PCBs-degrading capacity. We chose TG9 as a model environmental bacterium for the study of the VBNC state of PCBs-degrading bacteria. Through previous studies we found that cell wall changes may be a very important link in its entry into the VBNC state and resuscitation process, so we studied the cell wall changes of VBNC-state cells.

In the present study, *R. biphenylivorans* TG9 treated with 64 mg/L norfloxacin were induced into a state of VBNC after 7 days. The resuscitation of these cells was achieved by the removal of the inducing factor, namely the antibiotic. Notably, VBNC cells are easily confused with another dormant subpopulation of bacteria, antibiotic persister cells. In fact, these two kinds of cells are at different depth of dormancy. VBNC cells represent in a deeper dormancy state, which cannot grow on routine laboratory media even if inducing stress is removed. Antibiotic persister cells exist in an early state of dormancy, with the ability of growing on nutrient media after removal of antibiotics (Ayrapetyan et al., 2018). As shown in Figure 1A, the cultured cells counts decreased to an undetectable level after 5 days of norfloxacin treatment, which indicated there were no cells in the system that could grow on nutrient media. In other words, there were no antibiotic persister cells after 5 days of norfloxacin treatment. Besides, the number of viable cells almost no longer decreased at 7 days. Therefore, the norfloxacin-tolerant viable cells that remained after 7 days of treatment were VBNC cells rather than antibiotic persister cells.

During the entry of the VBNC state of the cells and the resuscitation of the cells, the cell wall thickness of TG9 changed. The cell wall of the VBNC-state TG9 cells was found to be thicker. Su et al. (2013) reported that the cell wall thickening of *Vibrio parahaemolyticus* in the VBNC state could have enhanced their tolerance to heat, H_2_O_2_, and low salinity. Moreover, it has been demonstrated that *Mycobacterium tuberculosis* cells under hypoxia develop a thick outer layer that helps restrict the entry of rifampicin (Jakkala and Ajitkumar, 2019). In our previous research, we found that the TG9 cells in VBNC-state were tolerant of oligotrophic and low temperature (Su et al., 2015b). Combined with our observation that the VBNC-state TG9 cells were tolerant to norfloxacin, we hypothesized that the thickening of the cell wall could be one of the reasons for the tolerance of these cells to norfloxacin. After the cells were resuscitated, the thickness of the cell wall was consistent with that of exponential cells. Although a thicker cell wall is an effective barriers, it also inhibits cell growth. Therefore, the remodeling of cell wall is indispensable for the growth resumption of dormant cells (Sexton et al., 2015).

Meanwhile, compared with the exponential phase cells and resuscitated cells, VBNC TG9 cells showed a significant increase in cell wall surface roughness. The roughness of bacterial cells can be affect by many factors, including the sample preparation. Therefore, we chose SEM and AFM to characterize the roughness of cells. The pretreatments of samples by these two methods were completely different. However, the results of SEM and AFM showed that the surface of TG9 cells in the VBNC state were rougher than that of exponential cells and resuscitated cells. In previous studies, the surface roughness of *Escherichia coli* cells after ampicillin treatment increased significantly, which may contribute to bacterial cells adhesion to model surfaces. Moreover, biofilm formation ability was also enhanced, promoting the survival of cells in adverse environments (Uzoechi and Abu-Lail, 2019). Therefore, the increase in cell wall surface roughness could be caused by the toxicity of norfloxacin, or may be one of the reasons for TG9 being tolerant to antibiotics in the VBNC state.

In addition, the electron microscopy images (Figures 2A–C, Figures 3A–C) revealed that VBNC cells still had intact cellular structure. Meanwhile, VBNC cells could be stained with CTC, showing that they were still viable and not ghost cells. The results of the resuscitation experiments (Figure 1B) also supported this point.

We also found that the genes related to the synthesis and remodeling of peptidoglycan were still maintaining high levels of expression during the entry of the VBNC state of TG9 cells. This result is consistent with previous observations of thickening of cell walls during cells entry into VBNC state. Furthermore, the structure of the glycan skeleton changed, correlating with previous reports. Signoretto et al. (2000) demonstrated that the expression of peptidoglycan biosynthesis-related gene was high in the VBNC-state *Enterococcus faecalis* cells. Meanwhile, the biosynthesis of penicillin binding proteins (1 and 5) involved in peptidoglycan assembly was enhanced (Lleò et al., 2007). Furthermore, previous studies have shown that the disturbance of cell wall synthesis dynamics are related to the formation of abnormal cells when *Vibrio parahaemolyticus* cells were induced to VBNC state (Hung et al., 2013). In our experiment, we found that the cell morphology of VBNC TG9 cells altered from the original rod shape to a coccoid shape. We speculated that these changes were also caused by changes in the synthesis kinetics of peptidoglycan.

In summary, our findings indicate that *Rhodococcus biphenylivorans* TG9 cells enter the VBNC state after treatment with norfloxacin and are resuscitated after the removal of the antibiotic. In addition, TG9 cells undergo changes in the thickness and roughness of their cell walls during their entry into the VBNC state and during their resuscitation, which is of great significance for the maintenance of antibiotic tolerance in this subpopulation. At the same time, changes in the synthesis kinetics of peptidoglycan caused by changes in the genes related to peptide polymerization and remodeling during entry into the VBNC state and the resuscitation of TG9 may account for the morphological changes. These findings are of great significance for the analysis of the role of the changes of cell walls in the entry of bacterial cells into the VBNC state and the process of resuscitation.

## Data Availability Statement

All datasets generated for this study are included in the article/Supplementary Material.

## Author Contributions

YJ: conceptualization, methodology, software, formal analysis, investigation, writing – original draft, and writing – review and editing. CY: methodology, investigation, and writing – review and editing. JF: validation, investigation, writing – review and editing. YF: validation, formal analysis, and writing – review and editing. ZY: methodology, software, and formal analysis. XG: methodology and formal analysis. YX: resources and funding acquisition. CS: conceptualization, methodology, resources, writing – review and editing, supervision, and funding acquisition. All authors contributed to the article and approved the submitted version.

## Conflict of Interest

The authors declare that the research was conducted in the absence of any commercial or financial relationships that could be construed as a potential conflict of interest.
