# Supplementary figures and images for: Alterations in the Cell Wall of Rhodococcus biphenylivorans Under Norfloxacin Stress
Source: Front Microbiol. 2020 Oct 6;11:554957. doi: 10.3389/fmicb.2020.554957 (PMC7573542; doi:10.3389/fmicb.2020.554957)

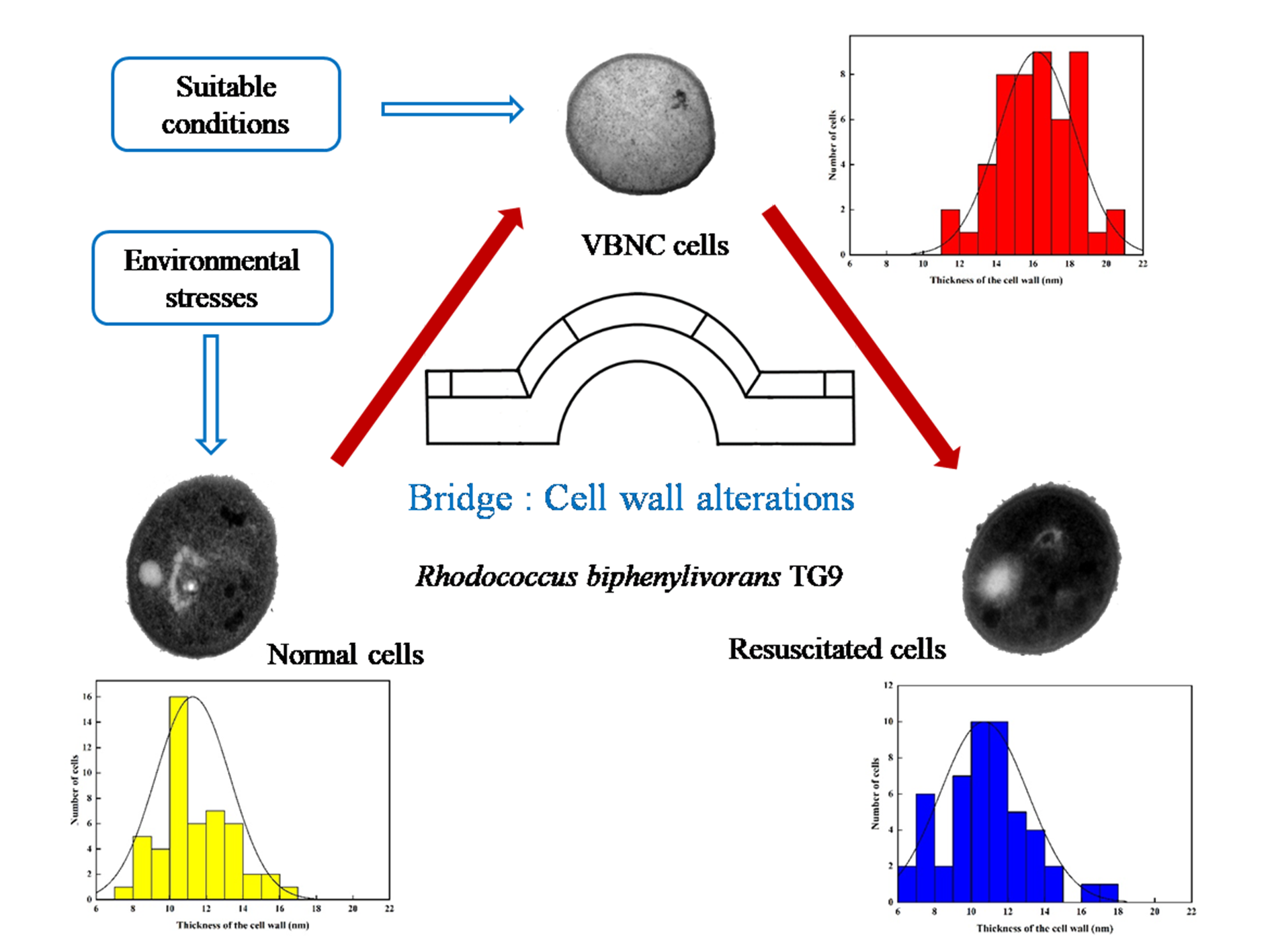

Supplement: Supplementary file 1 [file Image_1.PNG]
