# Supplementary material for: Alterations in the Cell Wall of Rhodococcus biphenylivorans Under Norfloxacin Stress
Source: Front Microbiol. 2020 Oct 6;11:554957. doi: 10.3389/fmicb.2020.554957 (PMC7573542; doi:10.3389/fmicb.2020.554957)
Supplement: Supplementary file 2 [file Data_Sheet_1.doc]

Supplementary Material

# Supplementary Figures and Tables

Table S. 1

Primers used in RT-qPCR experiments of this study

| Gene | Primer | **Sequence (5’ to 3’)** |
| --- | --- | --- |
| *16SrRNA* | Primer 1  Primer 2 | AAGTGACGGTAGTGGGAGAAGA  CAGTTGAGCTGCGGGATTT |
| *murA* | Primer 3  Primer 4 | ATCGTCGACCTGTGCAACAT  AATTTGTCCAGCACCAACGC |
| *ftsW* | Primer 5  Primer 6 | GGATCGGCCTACGGATTGTT  CAACGAGATACCGGCGATGA |
| *pknB* | Primer 7  Primer 8 | CAACGAGATACCGGCGATGA  GTAAGGACGTCCGTCGTGTT |
| *rpfE* | Primer 9  Primer 10 | AAAGTTCACGATCAAGCGCG  GACCGCCGTAATAGCCGTTA |

Figure S. 1

Figure S. 1 Muropeptide profile of TG9 cells in exponential phase obtained by LC/MS/MS

Figure S. 2

Figure S. 2 Muropeptide profile of TG9 cells in VBNC state obtained by LC/MS/MS
